# WAH-1/AIF regulates mitochondrial oxidative phosphorylation in the nematode *Caenorhabditis elegans*

**DOI:** 10.1038/s41420-017-0005-6

**Published:** 2018-01-29

**Authors:** Kostoula Troulinaki, Sven Büttner, Anaïs Marsal Cots, Simona Maida, Katharina Meyer, Fabio Bertan, Anna Gioran, Antonia Piazzesi, Alessandra Fornarelli, Pierluigi Nicotera, Daniele Bano

**Affiliations:** German Center for Neurodegenerative Diseases (DZNE), Bonn, Germany

## Abstract

Impaired mitochondrial energy metabolism contributes to a wide range of pathologic conditions, including neurodegenerative diseases. Mitochondrial apoptosis-inducing factor (AIF) is required for the correct maintenance of mitochondrial electron transport chain. An emerging body of clinical evidence indicates that several mutations in the *AIFM1* gene are causally linked to severe forms of mitochondrial disorders. Here we investigate the consequence of WAH-1/AIF deficiency in the survival of the nematode *Caenorhabditis elegans*. Moreover, we assess the survival of *C*. *elegans* strains expressing a disease-associated WAH-1/AIF variant. We demonstrate that *wah-1* downregulation compromises the function of the oxidative phosphorylation system and reduces *C*. *elegans* lifespan. Notably, the loss of respiratory subunits induces a nuclear-encoded mitochondrial stress response independently of an evident increase of oxidative stress. Overall, our data pinpoint an evolutionarily conserved role of WAH-1/AIF in the maintenance of proper mitochondrial activity.

## Introduction

Mitochondria contribute to energy production and biosynthetic processes in eukaryotic cells. Abnormal mitochondrial function contributes to the pathogenesis of age-dependent and familial forms of neurodegenerative disorders^1–5^. Furthermore, impaired maintenance of mitochondrial biogenesis, structure, and bioenergetics may lead to the development of a variety of human diseases, including cardiomyopathies, encephalomyopathies, rare metabolic syndromes, peripheral neuropathies, and liver failure. In this regard, a growing body of evidence supports the notion that altered oxidative phosphorylation (OXPHOS) is causally associated with several sporadic and inherited pathologies known as mitochondrial disorders^2,6–10^. Their clinical manifestations range from limited damage of a single tissue to devastating systemic failure of various organs. In most of the inherited forms, mitochondrial disorders exhibit a chronic and progressive course with an onset that often occurs perinatally or within the first years of life^9,11–13^. The pathophysiology of mitochondrial disorders is challenging, as they may be due to mutations either in nuclear- or mitochondrial DNA-encoded genes and can directly or indirectly affect the OXPHOS system. To make diagnostic and therapeutic interventions even more difficult, different mutations in a single gene can give rise to distinct syndromes, whereas a large spectrum of pathogenic mutations in various genetic loci can lead to an identical clinical outcome, as in the case of Leigh syndrome^7,10,14^. Despite the relatively low incidence within the population^8,15^, an increasing number of new disease-causing mutations have been isolated in patients with mitochondrial disorders.

Apoptosis-inducing factor (AIF) is a FAD-containing protein with an NADH-dependent oxidoreductase activity^16,17^. It was initially described as a death effector that is released from mitochondria in response to toxic insults^16,18^. Its pro-apoptotic role is highly conserved from yeast to invertebrates to mammals^19^. The mature form of AIF is mainly tethered to the mitochondrial inner membrane^16,20,21^. Structural and biochemical characterizations of in vitro purified AIF show that NADH incorporation determines the folding, the redox status, and the dimerization of the FAD-containing AIF protein^22–25^. In addition to its death-related role, AIF also contributes to cellular bioenergetics, as it assists in the assembly and/or stabilization of the electron transport chain (ETC)^26^. In this regard, our group and others have demonstrated that AIF physically interacts and stabilizes the oxidoreductase CHCHD4/MIA40, hence assisting the correct biogenesis of the respiratory chain complexes^27,28^. Over the last years, linkage analysis and exome sequencing of patients have identified an array of deleterious mutations in the *AIFM1* gene that are associated with rare inherited X-linked mitochondrial disorders. The first deleterious mutation in the *AIFM1* gene was found in two infant males showing progressive mitochondrial encephalomyopathy and born from twin sisters^29^. The disease-segregating mutation is an inherited trinucleotide deletion in exon 5 that causes the ablation of the Arg201 (R201 del). As a result, recombinant mutant AIF protein is structurally unstable, shows aberrant FAD incorporation, and, consequently, impaired redox properties^22,29^. To date, a significant array of mutations in the *AIFM1* gene have been identified in patients showing a wide range of clinical presentations^17,22,30–38^.

The Worm AIF Homolog WAH-1 is a mitochondrial protein that is released into the cytosol during apoptosis. In association with CPS-6/Endonuclease G, WAH-1 translocates to the nucleus and contributes to DNA degradation during caspase-dependent cell death^39^. Despite the presence of putative FAD- and NADH-binding domains, it seems that WAH-1 does not incorporate cofactors and undergoes conformational changes according to the redox status of the surrounding milieu. At least in vitro, oxidized WAH-1 forms inter-molecular disulphide bonds and exists as dimers or high-order oligomeric states, whereas it dissociates to monomers in response to reduced conditions^40^. Apart from development, the importance of WAH-1 in other physiological contexts, including mitochondrial activity, has not been fully addressed yet. Here we investigate the downstream consequences of WAH-1/AIF genetic modulation in *C*. *elegans*. We demonstrate that *wah-1* downregulation by RNA interference (RNAi) affects the expression of ETC complexes and mitochondrial respiration, resulting in reduced lifespan. Moreover, we present new CRISPR/Cas9-modified knockin strains in which a disease-associated WAH-1 variant is expressed. Our data indicate that, along with its well-established role in cell death, WAH-1/AIF is required for the correct maintenance of the OXPHOS system in the nematode *C*. *elegans*.

## Results

### WAH-1 deficiency shortens *C. elegans* lifespan by altering mitochondrial activity

**Fig. 1:**
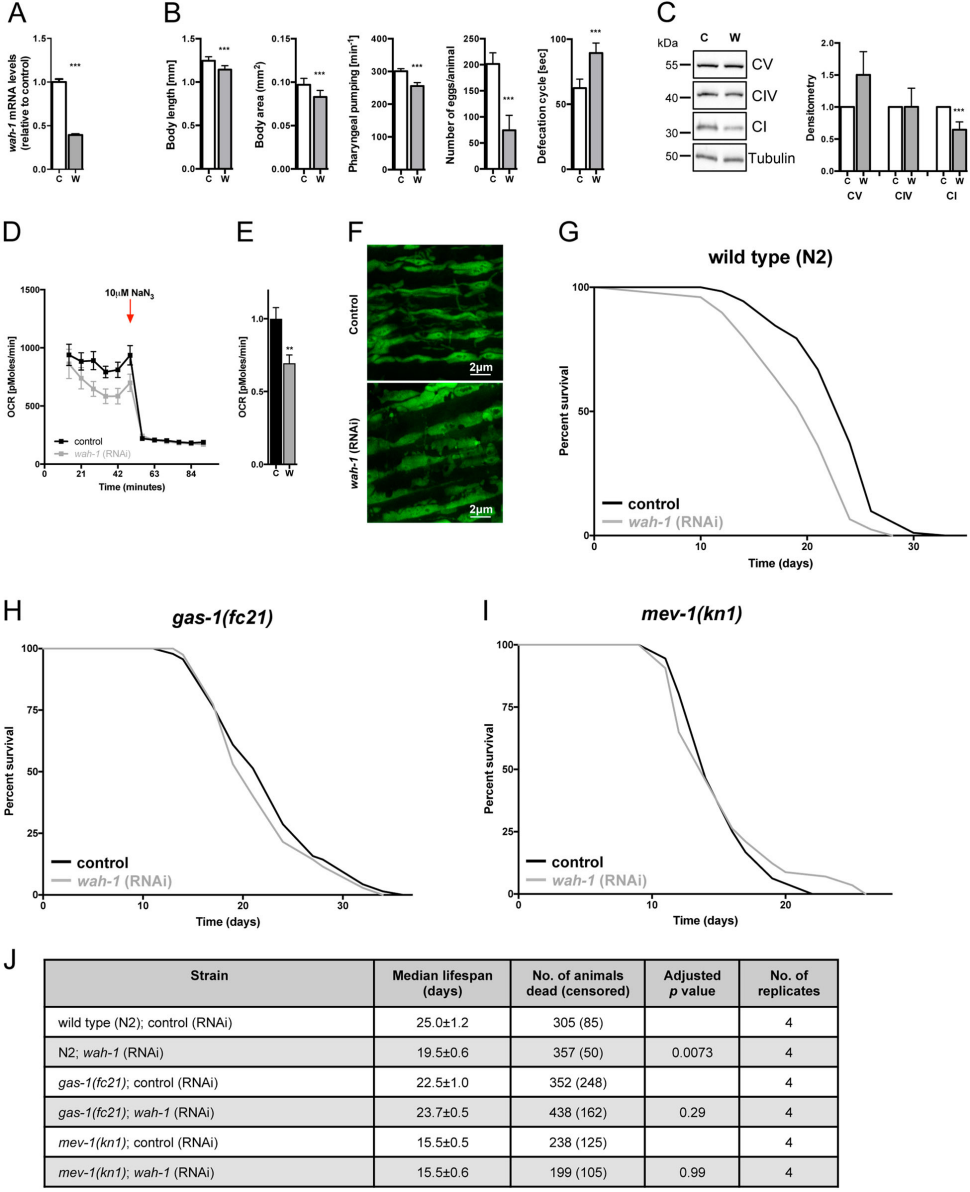
WAH-1 deficiency shortens *C. elegans* lifespan and affects mitochondria **a** *wah-1* mRNA levels in 4-day-old animals fed with control and *wah-1* RNAi. **b** Phenotypic analyses of *wah-1*-silenced animals show differences in body length, body area, pharyngeal pumping, egg laying, and defecation rate. **c** Knockdown of *wah-1* destabilizes the ETC in wild-type nematodes. Densitometric analysis is reported in the lower panel (*n* > 3). **d** WAH-1 deficiency reduces OCR. NaN3 was used as mitochondrial respiration inhibitor. **e** Statistical analysis of basal respiration in nematodes grown on control and *wah-1* RNAi bacteria (**p < 0.01). **f** STED microscopy of nematodes expressing mitochondria-targeted GFP reveals altered organelle morphology in the muscle of animals grown on *wah-1* RNAi. **g** *wah-1* knockdown reduces the lifespan of wild-type animals. **h**, **i** Knockdown of *wah-1* does not affect the lifespan of **h** complex I mutant *gas-1(fc21)* as well as of **i** complex II mutant *mev-1(kn1)*. **j** Lifespan table relative to experiments in Fig. 1g-i. Survival curves represent the lifespan of nematode populations: *y*-axis shows the percentage of animals alive, *x-*axis shows the time in days; black: control, light gray: *wah-1* RNAi

To determine whether WAH-1/AIF is an essential protein for *C*. *elegans* physiology, synchronized wild-type N2 nematodes were exposed to control and *wah-1* RNAi. Quantitative real-time PCR confirmed *wah-1* downregulation (Fig. 1a). Consistent with prior findings^39^, WAH-1 deficiency affected growth rate, body size, pharyngeal pumping, fertility, and defecation rhythm (Fig. 1b). As AIF contributes to the correct maintenance of the OXPHOS system in mammals^26–28^, we performed immunoblot analysis of ETC subunits and observed decreased complex I subunit NUO-2/NDUFS3 expression in *wah-1*-silenced nematodes (Fig. 1c). Consistently, oxygen consumption rate (OCR) was reduced in *wah-1* deficient nematodes, further indicating an impaired mitochondrial activity (Figs. 1d-e). We examined mitochondrial morphology in transgenic lines carrying a mitochondria-targeted green fluorescent protein (GFP) driven by the muscle-specific *myo-3* promoter. Stimulated emission depletion (STED) super-resolution microscopy analysis showed mitochondria with an irregular appearance already in young WAH-1 deficient animals (Fig. 1f). We examined the lifespan of *wah-1*-silenced nematodes and found a reduction in their median lifespan compared to controls (Fig. 1g, j). This effect is independent of development, as *wah-1* RNAi treatment only during adulthood still shortened nematode lifespan (data not shown). To test whether WAH-1 deficiency regulates the lifespan through the ETC, we performed epistasis analysis using *gas-1(fc21)* and *mev-1(kn1)* mutants. The *gas-1* gene encodes for a 49 kDa subunit of complex I, whereas the *mev-1* encodes for a subunit of complex II. We found that *wah-1* RNAi did not alter the median lifespans of neither *gas-1* nor *mev-1* mutant animals (Figs. 1h-j), further supporting that lifespan reduction due to WAH-1 deficiency is dependent on mitochondrial function. Taken together, our findings demonstrate the evolutionarily conserved role of WAH-1 in the assembly and/or stabilization of the mitochondrial ETC.

### WAH-1 downregulation induces mitochondrial stress signaling

**Fig. 2:**
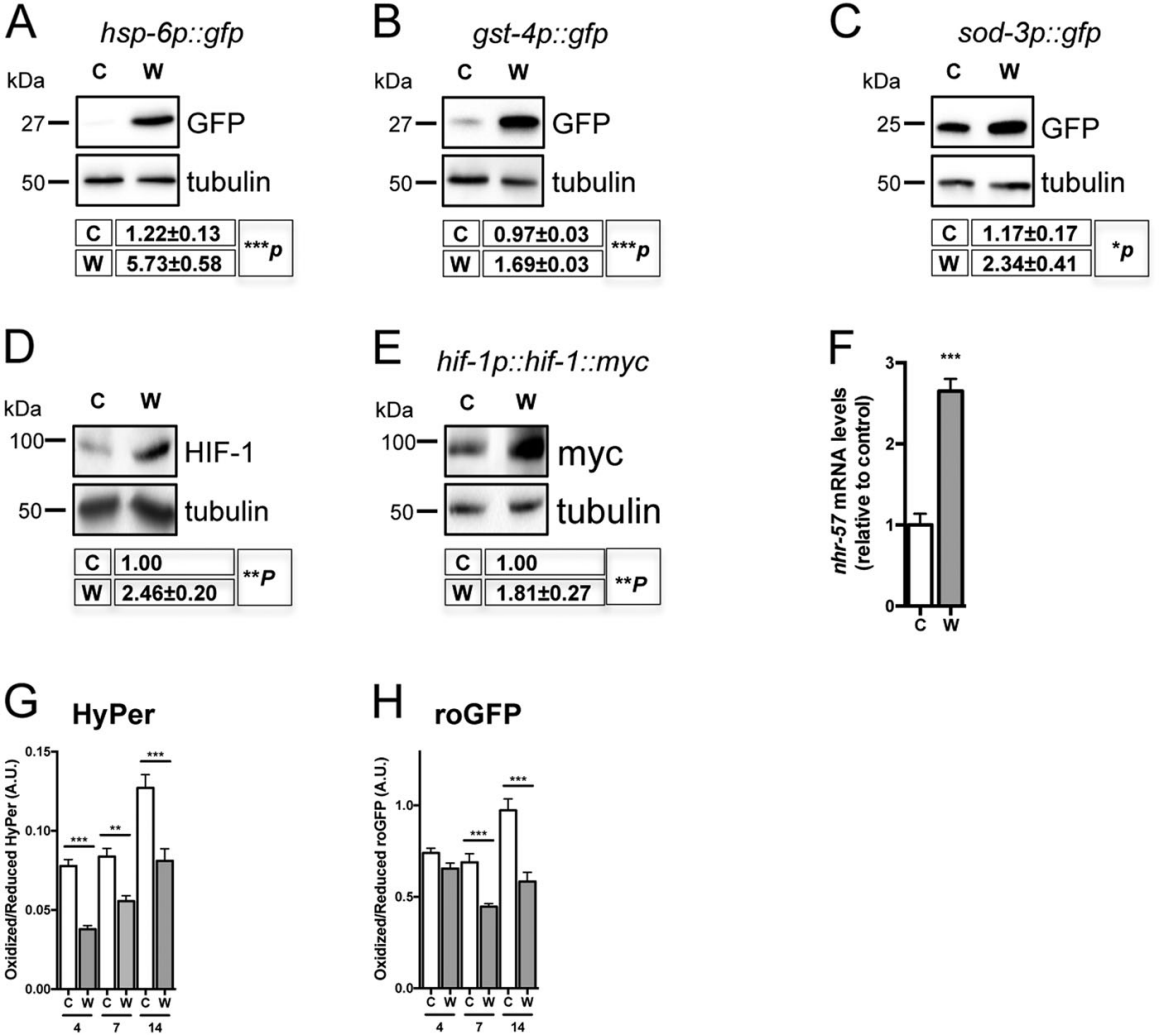
WAH-1 downregulation triggers nuclear-encoded stress response **a**–**c** Immunoblot analyses show GFP expression driven by **a** hsp-6, **b** *gst-4* and **c** *sod-3* promoters in *C*. *elegans* strains grown on control and *wah-1* RNAi. **d**, **e** *wah-1* downregulation stimulates the accumulation of **d** endogenous HIF-1 and **e** Myc-tagged HIF-1 (*n* > 4). **f** qRT-PCR shows upregulation of the HIF-1-target gene *nhr-57* in *wah-1*-silenced nematodes compared with control ones. **g**, **h** *wah-1* downregulation results in decreased ROS levels as assessed with **g** the peroxide-specific sensor Hyper (*n* = 2) and **h** the redox sensitive biosensor roGFP (*n* = 2) (for all experiments, mean ± SEM, ***p < 0.001, **p < 0.01, *p < 0.05, two-tailed Student’s *t-*test or ordinary one-way ANOVA). Abbreviations: C, control; CI, complex I; CIV, complex IV; CV, complex V; W, *wah-1* RNAi

In response to mitochondria-induced stress, eukaryotic cells robustly upregulate the expression of nuclear-encoded detoxifying systems^41^. Consistently, we found that *wah-1* downregulation activated *hsp-6* (heat-shock-protein-6), *gst-4* (glutathione-*S*-transferase), and *sod-3* (superoxide dismutase) promoters (Fig. 2a-c), further suggesting an enhanced mitochondrial stress response. Inhibition of mitochondrial respiration activates the hypoxia-inducible factor HIF-1^42^. We assessed HIF-1 protein levels and activity in *wah-1*-silenced animals and detected a significant increase of HIF-1 protein level (Fig. 2d, e), which was associated with an enhanced expression of the HIF-1 target gene *nhr-57* (Fig. 2f). In many mitochondrial mutant nematodes, altered mitochondrial respiration can enhance oxidative stress and induce the formation of free radicals^43^. Thus, we assessed reactive oxygen species (ROS) levels in *wah-1*-silenced animals. Surprisingly, nematodes fed with *wah-1* double-stranded RNA (dsRNA)-expressing bacteria showed decreased levels of ROS as measured with the two genetically encoded fluorescent biosensors Hyper and roGFP^44^ (Fig. 2g, h). Taken together, these findings demonstrate that WAH-1 loss alters mitochondrial respiration and triggers a mitochondria-to-nucleus response independently of ROS signaling.

### WAH-1 knockin variants have a negligible impact on *C. elegans* lifespan

**Fig. 3:**
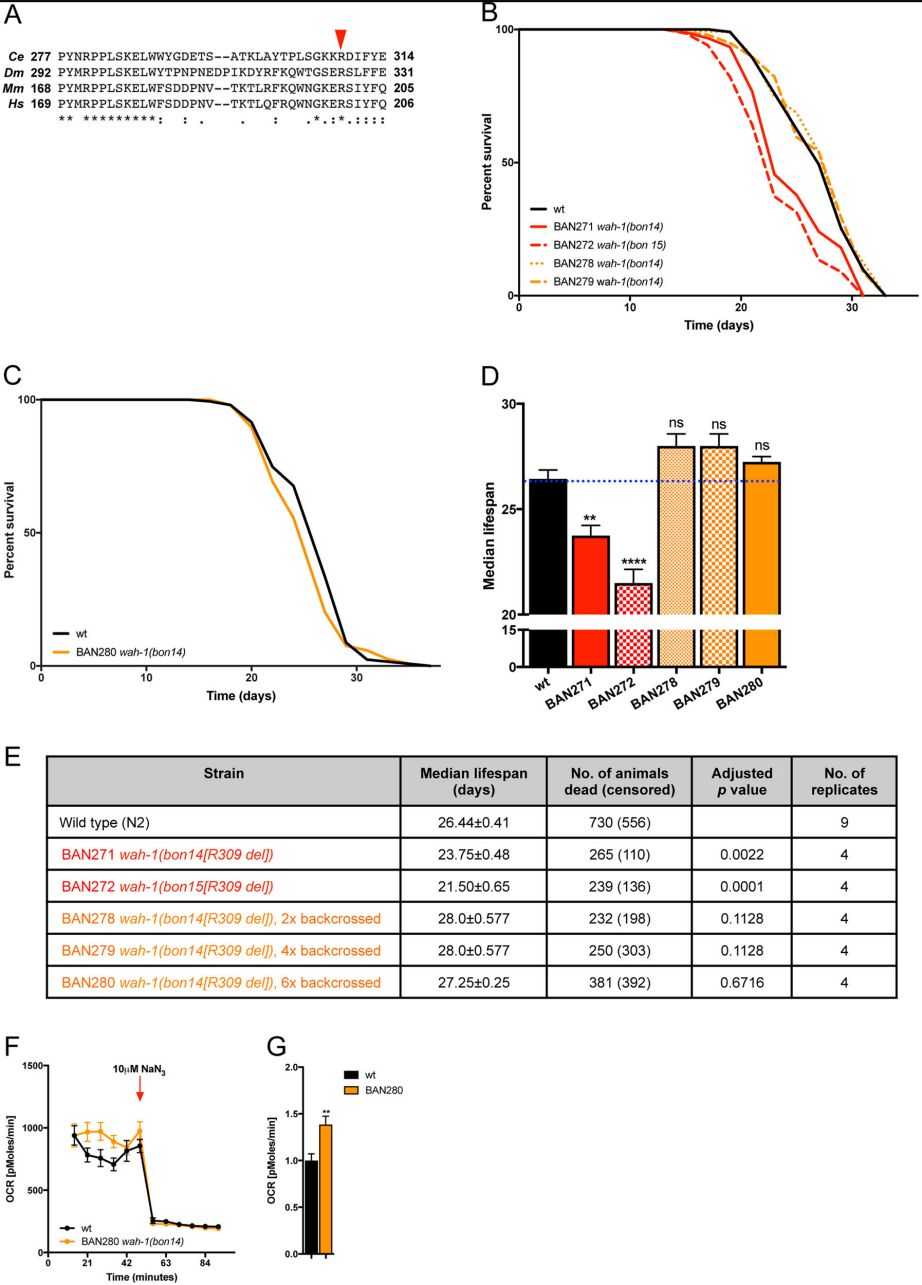
AIF disease-causing mutation has limited effect on *C. elegans* lifespan **a** Multiple amino acid alignment of WAH-1/AIF region consisting of Pro277 to Glu314. The human Arg201 (red arrow) is conserved across species. **b**–**c** Lifespan assay of wild-type (black line) and nematode expressing WAH-1 (R309 del) variant (red and orange lines). Although BAN271 and BAN272 show a reduced lifespan of a few days, outcrossed animals have the same lifespan as wild-type ones. Solid black line, wild-type N2; red solid line, BAN271; red dashed line, BAN272; orange dotted line, BAN278; orange dashed line, BAN279; orange solid line, BAN280. **d** Cumulative lifespan experiment plot of wild type and WAH-1 mutant animals (**p < 0.01, ****p < 0.0001; NS, nonsignificant). **e** Lifespan table relative to experiments in Fig. 3b-c. **f** OCR in wild type and WAH-1 (R309 del) mutant nematodes. **g** Statistical analysis of basal respiration of wild type and WAH-1 (R309 del) mutant nematodes. NaN3 was used to inhibit mitochondrial respiration (**p < 0.01)

Deletion of Arg201 results in an unstable AIF protein and, consequently, human pathology^29^. Based on amino acid sequence alignment, Arg201 is conserved across the animal kingdom. The *C*. *elegans* homolog is Arg309 and resides within a highly conserved motif that regulates FAD incorporation in the mammalian AIF (Fig. 3a). To determine the physiological impact of AIF variants in vivo in nematodes, we obtained two independent lines in which the endogenous *wah-1* locus was modified using CRISPR/Cas9 gene editing. We assessed the lifespan of these original strains as well as the outcrossed ones. Although the original mutants showed a significant lifespan reduction, we found that outcrossed animals did not exhibit any difference in survival (Fig. 3b-e). We measured basal respiration and, contrary to *wah-1*-silenced animals, WAH-1 (R309 del) mutants showed an unexpected increase, rather than a decrease, of oxygen consumption compared with wild-type nematodes (Fig. 3f, g). Together, these findings show that the WAH-1 (R309 del) variant has no effect on *C*. *elegans* survival and does not impair mitochondrial respiration.

## Discussion

AIF is a mitochondrial protein essential for the maintenance of the OXPHOS system. A significant number of *AIFM1* mutations have been identified in patients with mitochondrial disorders, further underlying the importance of AIF in human pathophysiology. With the aim of generating new in vivo models of AIF deficiency, here we describe AIF-mediated mitochondrial impairment in the nematode *C*. *elegans*. We show that WAH-1 downregulation affects mitochondrial morphology, induces ETC dysfunction and reduces the respiration rate. Consistently, epistasis experiments in mutants carrying mitochondrial defects show that *wah-1* silencing affects lifespan by impairing mitochondrial respiration. In an effort to study *AIFM1* disease-causing mutations, we obtained CRISPR/Cas9-modified nematodes expressing a disease-associated WAH-1/AIF variant. Contrary to the clear effect of *wah-1* silencing, outcrossed WAH-1 (R309 del) animals do not show any difference in survival compared to wild type nematodes. To a certain extent, WAH-1 modulation of mitochondrial function and survival is consistent with the effect of AIF in higher organisms. Indeed, defects of the OXPHOS system varies considerably according to the level of AIF protein loss, with the complete AIF knockout that compromises multiple OXPHOS subunits, whereas AIF downregulation determines mainly aberrant complex I activity^26–29,45,46^. As in mammals, it may be that WAH-1 controls the OXPHOS system through the interaction with the four CHCHD4/MIA40 orthologs encoded in *C*. *elegans*. Alternatively, WAH-1 may modify the electron flow through the respiratory chain complexes, promoting proton pumping across the mitochondrial membrane in a similar manner to the yeast NADH-ubiquinone oxidoreductase Ndi1 as recently proposed^47,48^. Ultimately, WAH-1 may bind factors that directly regulate mitochondrial biogenesis. In this regard, it is known that the redox state of WAH-1 affects the activity of CPS-6/EndoG^40^. As CPS-6 and EndoG mediates the depletion of mitochondrial DNA in nematodes and mammals, respectively^49,50^, it may be that the archaic WAH-1-dependent regulation of the OXPHOS system is functionally linked to the mitochondrial DNA homeostasis. Further work is necessary to elucidate this biological aspect.

It is worth noting that, although severe mitochondrial dysfunction contributes to several human pathologies, mild mitochondrial uncoupling or modest reduction of OXPHOS during development promotes longevity in some model organisms^43,51,52^. Multiple lines of evidence suggest that the respiration rate induces metabolic changes, favoring alternative survival pathways as well as protective homeostatic mechanisms^43,53–55^. Moreover, mild mitochondrial stress induces the expression of nuclear-encoded genes through epigenetic modifications of chromatin, including posttranslational modification of histones and nucleosome turnover^56–59^. Thus, inhibition of mitochondrial respiration can mediate stress response and adaptive processes with a wide array of homeostatic functions^43,60^, although the engagement of this complex surveillance pathway is not a predictor of *C*. *elegans* lifespan^61^. In our study, we demonstrate that nematodes subjected to *wah-1* RNAi exhibit a nuclear stress response that includes the upregulation of *hsp-6*, *sod-3*, *gst-4*, and HIF-1 target genes. However, such a transcriptional response is insufficient to counteract OXPHOS defects upon *wah-1* knockdown, contrary to other genetic manipulations^51,62^. As a result, *wah-1* downregulation shortens, rather than extends, *C*. *elegans* lifespan. One reasonable possibility that explains the decreased survival is an enhanced level of ROS due to ETC impairment. Perhaps surprisingly, multiple approaches indicate that animals lacking WAH-1 exhibit much lower ROS levels compared with control ones. At least in vivo, it is unlikely that WAH-1 has an antioxidant function or a free radical-scavenging activity, although we do not rule out that it contributes to redox reactions through direct interaction with other proteins as previously shown^23,24,40^. Our data further support the concept that increased oxidative stress in certain AIF-deficient models may be a consequence, rather than the cause, of mitochondrial dysfunction. Moreover, our findings demonstrate the conserved role of WAH-1/AIF in the correct maintenance of a functional OXPHOS system in the nematode *C*. *elegans*.

## Materials and methods

### Antibodies

The following antibodies were obtained from commercial sources as indicated: mouse anti-Myc (Cell Signaling Technology); rabbit and mouse anti-GFP (Invitrogen and Roche); mouse anti-ATP synthase subunit alpha (CV), mouse anti-complex IV subunit 1, mouse anti-complex I subunit NDUFS3 (CI), mouse anti-complex I subunit NDUFA9 (CI) (Mitosciences, Abcam); mouse anti-actin, mouse anti-tubulin (Sigma). Secondary horseradish peroxidase-conjugated anti-mouse and anti-rabbit antibodies were purchased from Pierce.

### *C. elegans* methods and strains

Nematode strains were maintained on Nematode Growth Medium (NGM) plates seeded with *Escherichia coli* strain OP50 or HT115 (DH3) as a food source and kept at 20 °C as described previously^63–65^. The following strains were used: N2 (wild type, Bristol isolate), BAN271 *wah-1(bon14[R309 del])*, BAN272 *wah-1(bon15[R309 del])*, BAN278 *wah-1(bon14[R309 del])* 2 × backcrossed, BAN279 *wah-1(bon14[R309 del])* 4 × backcrossed, BAN280 *wah-1(bon14[R309 del])* 6 × backcrossed, CF1553 *muls84*[*pAD76(sod-3::*GFP*)*], CL2166 *dvIs19*[*pAF15(gst-4::*GFP::NLS*)*], CW152 *gas-1(fc21)X*, *jrIs1[Prpl-17::Hyper]*, *jrIs2 [Prpl-17::Grx-1-roGFP2]*, SJ4100 *zcIs13*[*hsp-6::*GFP], SJ4103 *zcIs14*[*myo-3p::*GFP(mit)], TK22 *mev-1(kn-1)III*, and ZG580 *iaIs28*[*hif-1p::hif-1a::tag*]. The following strains were obtained from Knudra Transgenics: BAN271 *wah-1(bon14[R309 del])* and BAN272 *wah-1(bon15[R309 del])*.

### Lifespan assays and RNAi

Unless otherwise stated, all experiments were conducted at 20 °C. RNAi was performed using a bacterial feeding protocol. Briefly, adult gravid nematodes were treated with hypochlorite solution to obtain synchronous populations. Bleached eggs were rinsed with M9 buffer and immediately transferred to NGM plates carrying *E. coli* HT115 bacteria expressing dsRNAs against target genes (Ahringer library, Source Bioscience LifeSciences). The expression of dsRNA was induced by isopropyl-β-d-1-thiogalactopyranoside (1 mM). A bacterial strain carrying the empty pL4440 vector served as control. Progeny was grown on NGM plates until adult stage and then transferred to fresh plates at groups of 30–45 nematodes per plate. Animals were transferred to fresh plates every 1–4 days and examined every second day for touch-provoked movement and pharyngeal pumping, until death. Worms that died abnormally due to internally hatched eggs, protruded vulva or dried on the edge of the plates were considered censored. Survival curves were generated with GraphPad Prism Software (GraphPad Software Inc., San Diego, USA) using the method of Kaplan and Meier. *p*-values were calculated using the log-rank (Mantel–Cox) test.

### Microscopy

Nematodes were paralyzed with 25 mM levamisole in M9 buffer mounted on 2 % agarose pads or immobilized with 30 % PEG/25 % glycerol solution on glass slides. Alternatively, animals were fixed on 5 % agarose pads with a solution of polybead polystyrene (0.10 μm microspheres) (Polysciences). For quantification, we used Image J software unless noted otherwise. Statistical analysis was performed with GraphPad Prism Software and *p*-values were determined using *t*-test or analysis of variance. *Stimulated emission depletion microscopy*: STED microscopy was performed with an inverted microscope (Leica DMI6000 CS) equipped with a × 100 oil objective and a white light laser. Images were taken at a resolution of ~50 nm following the use of a depletion laser at 592 nm. *ROS quantification by microscopy: f*or the quantification of H_2_O_2_ in *jrIs1 [Prpl-17::Grx-1-Hyper]* and GSSG/2GSH ratios in *jrIs2 [Prpl-17::Grx-1-roGFP2]* animals, an Andor Spinning Disk setup was used. The system consisted of a fully motorized inverted Nikon microscope in association with a Yokogawa Spinning Disk connected to a back-illuminated EMCCD camera (Andor iXON DU-897, 512 × 512 pixels, 16 bit, 35 frames per second). The setup was equipped with a × 40 oil-immersion lens (Nikon). Fluorescence was measured with a 525 nm emission filter, exciting the probes with 405 and 488 nm excitation lasers. Animals were analyzed at 4, 7, and 14 days of age.

### OCR measurement

OCR was measured using the Seahorse XF24 Analyzer (Agilent, Copenhagen, Denmark). Nematodes were grown for 4 days on control and *wah-1* (RNAi), washed with M9 buffer and transferred into XF24 Cell Culture Microplates at a final number of 50 animals per well. Nematodes were left to equilibrate at 20 °C for 90 min, then transferred to the Seahorse machine. OCR was recorded by the Seahorse XF24 Software under basal conditions as well as in response to 10 mM sodium azide (NaN_3_). Statistical analysis was performed using the GraphPad Prism Software package (GraphPad Software Inc.) and *p-*values were determined using a *t*-test.

### Quantitative real-time PCR

RNA extraction, purification and reverse transcription were performed by using the QIAshredder, RNeasy RNA extraction kit (Qiagen), and qScript cDNA Supermix (Quanta Biosciences). Quantitative RT-PCR was carried out in a Step One Plus Real Time PCR System (Applied Biosystems) and analyzed using the comparative ΔΔCt method. mRNA levels of β-actin were used for normalization. The average of at least three technical repeats was used for each biological data point. The following oligonucleotides were used in this study: *beta-actin* 5′-tgtgatgccagatcttctccat-3′ and 5′-gagcacggtatcgtcaccaa-3′; *wah-1* 5′- gctgatgctgtcgaggaga-3′ and 5′-tggtggtgttctcttctgtaga-3′; *nhr-57* 5′-tcggaatgaatccggaagt-3′ and 5′-atgcaggggaagatgaacag-3′.

### SDS gel electrophoresis and western blot analysis

Approximately 200 nematodes were collected in 1.5 ml sample tubes and resuspended in RIPA buffer. Mechanical disruption was achieved by sonication and total lysate was quantified with Bradford reagent (Sigma). Samples were denatured in Laemmli buffer at 95 °C for 5 min and spun down at 10,000 *g* for 10 min. Proteins were separated on 10–15 % poly-acrylamide gels and subsequently transferred onto nitrocellulose membranes using a wet blot chamber or a semi-dry blot cassette (Bio-Rad). Nitrocellulose filters were saturated in Tris Buffer Saline with 5 % milk powder and 0.05 % Tween-20 for 2 h at room temperature. Primary and secondary antibodies were incubated for 1 h at room temperature or overnight at 4 °C in blocking solution. Immunoblots were developed in ECL and images taken with the chemiluminescent analyzer Chemidoc imaging system (Bio-Rad).
